# Functional improvement of natural *Saccharomyces cerevisiae* yeast strains by cell surface molecular engineering

**DOI:** 10.1186/s13062-025-00614-1

**Published:** 2025-02-14

**Authors:** Sara Granuzzo, Monica Rossetto, Lucio Zennaro, Francesca Righetto, Paolo Antoniali, Raffaele Lopreiato

**Affiliations:** 1 Department of Biomedical Sciences, University of Padova, via U.Bassi 58b, Padova, 35131 Italy; 2 Department of Molecular Medicine, University of Padova, via Gabelli 63, Padova, 35121 Italy; 3 Italiana Biotecnologie Srl, via Vigazzolo 112, Montebello Vicentino, 36054 VI Italy

**Keywords:** *Saccharomyces cerevisiae*, Yeast surface display, Protein engineering, Bioremediation

## Abstract

**Background:**

Cellular boundaries of microorganisms can be modified by the expression in the cell wall of specific proteins endowed with relevant properties, improving their functional performance. So far, the surface display (SD) technique had been widely employed in the yeast *Saccharomyces cerevisiae*, but it was limited to few laboratory strains and never explored in *sauvage* strains, i.e., isolated from natural environment, which are featured by higher levels of genetic variability, leading to peculiar phenotypic traits of possible advantage in biotechnology.

**Results:**

In this work, a series of plasmids performing SD in natural yeast strains have been generated and further characterized by multiple functional and biochemical assays, providing the first experimental evidence that natural strains of *S.cerevisiae* can be genetically modified to express on their cell wall a protein-of-interest, which retains its biological competence. Interestingly, data further demonstrated that engineered strains expressing (transiently or stably) metal-binding proteins or peptides on cell surface exhibit significantly enhanced metal adsorption properties.

**Conclusions:**

The molecular tools presented here can be very useful for yeast research community, as the plasmids efficiently support the surface engineering in virtually all *S.cerevisiae* strains, independently from either genetic background, source, or applications (wine, beer, bread). Overall, data strongly suggest that, upon genetic modification, *S. cerevisiae* strains isolated from natural environments could serve as promising platforms for biotechnological applications, as heavy metals removal or enzymes immobilization. Importantly, the strains investigated here represent only a small fraction of the multitude of *S. cerevisiae* strains present in nature yet to be isolated.

**Supplementary Information:**

The online version contains supplementary material available at 10.1186/s13062-025-00614-1.

## Introduction

“Surface Display” is a genetic engineering technique so long applied in various microorganisms (bacteria, yeasts, algae), that enables the cells to produce a specific protein-of-interest by exposing it on the outer surface, where becomes stably associated with the cell wall [1]. The first application in *Saccharomyces cerevisiae* date back to 25 years ago [2], but over time the “Yeast Surface Display” (YSD) strategy allowed to express on the yeast cell wall a multitude of peptides and proteins (orthologous and heterologous) endowed with very different biological properties, as antibodies and nanobodies [3], metal-binding proteins [4–9], and also monomeric enzymes or multienzymatic complexes (i.e., biocatalysts) [10–13]. Importantly, *S.cerevisiae* is one of the most widely used species in human applications, ranging from food preparation (bread, wine, beer) to biotechnology, including the production of bioethanol and pharmaceuticals (e.g., insulin) [14]. Classified as GRAS organism (Generally Recognized as Safe), *S. cerevisiae* has a fast growth rate and minimal nutritional requirements, making it possible to produce large quantities of biomass at low cost. These factors make the use of YSD engineered yeast cells a highly promising (and sustainable) alternative for multiple applications, e.g., as immobilization platforms for specific biocatalysts, or as biological agents for heavy metals decontamination.

Generally, any protein expressed on the yeast cell surface must include at least two specific structural elements: a secretion signal, which directs the protein through the secretory pathway, and a cell wall anchoring domain, which associates the protein with the yeast cell wall. The secretion signal commonly used in YSD studies with *S. cerevisiae* [15] is the α-pheromone sequence (encoded by the *MFα1* gene), produced by α-cells during mating [16]. Various sequences have been employed as surface anchor domains, particularly when considering the topology of the chimeric protein (i.e., amino- or carboxy-terminal fusion). The primary C-terminal targeting strategies involve the Aga2p sequence, which enables covalent bonding (via S-S bridges) between the chimera and the Aga1p cofactor, a protein natively located in the cell wall [17]. N-terminal anchoring sequences are derived from proteins such as flocculation-related Flo1p protein [18], or Sag1p, an α-agglutinin [15]. Both proteins contain glycosylphosphatidylinositol (GPI) anchoring sequences that help localize the proteins within the cell wall [15].

Regardless of the chosen strategy, both molecular composition and remodeling of the cell wall are critical for determining the expression levels of fusion proteins on the yeast surface. The cell wall is a rigid structure primarily composed of β-linked glucans and mannoproteins [6]. However, it is also dynamic, with proteins being inserted in a temporally and spatially controlled manner. These processes are closely linked to pathways involved in cell wall biogenesis, such as those regulating the cell cycle and mating [19, 20]. Beyond surface remodeling, the production and stability of chimeric proteins on the cell surface are influenced by the yeast’s secretory properties. Chimeric proteins reach the cell wall via the classical secretory pathway, passing through the endoplasmic reticulum (ER), Golgi apparatus, and vesicular transport system, where posttranslational modifications like glycosylation occur [21]. Unsurprisingly, cell wall biogenesis and secretion are complex processes regulated by multiple genes [22, 23], as indicated by some *S. cerevisiae* mutants exhibiting enhanced protein secretion efficiency [24, 25].

To date, however, the YSD system has only been explored using laboratory-derived strains of *S. cerevisiae*, which represent a small, domesticated subset with features that are ideal for research purpose. Moreover, most experimental analyses have focused on strains derived from common ancestors, sharing similar phenotypes. Conversely, next-generation sequencing (NGS) of many yeast strains has revealed that those isolated from natural environments, such as those used in the industrial production of beverages like wine and beer, display high genomic variability [26–29]. Unique combinations of variant alleles may confer specific traits to natural *S. cerevisiae* strains, as reported for some industrial strains showing greater resistance to heat shock or oxidative stress compared to laboratory strains [30].

As mentioned, no studies have so far explored the use of natural *S. cerevisiae* strains for possible YSD-based applications. This gap was likely due to the absence of suitable molecular tools (e.g., plasmids for protein expression or CRISPR/Cas9 genome editing), and the unique challenges posed by manipulating natural yeast cells (e.g., selection markers and transformation protocols). Yet, natural environments harbor diverse *S. cerevisiae* strains with unique traits, shaped by selective pressures. Many of these strains have been isolated and selected for various technological applications. As previously suggested, new opportunities may arise from the identification of yeast variants with better performance than current strains [31].

Main goal of this work therefore consisted in the generation of new molecular tools, able to support the YSD strategy in natural *S. cerevisiae* strains, which may be useful for the entire researcher’s community. Experimentally, some natural strains from different backgrounds were genetically modified and then analyzed by biochemical and functional assays, to monitor both expression and cell wall localization of three different heterologous proteins. Additional data further confirmed that yeast cells expressing metal-binding chimeric proteins significantly increased their ability to reversibly sequester heavy metals (nickel and copper) from contaminated solutions. These findings suggest that natural *S. cerevisiae* strains, upon surface engineering, could have unprecedented applications in bioremediation.

## Materials and methods

### Strains and media

Yeast (*Saccharomyces cerevisiae*) and bacterial (*Escherichia coli*) strains used in this work are described in Supplementary Material and Table ST1. As previously reported [32, 33], yeast strains were maintained in standard complete medium (YPD, Yeast extract 10 g/L, Bacto Peptone 10 g/L, glucose 20 g/L), eventually supplemented with antibiotics (Nurseothricin or G418 Geneticine, 0.1 g/L) for selection. Synthetic SD medium (1.7 g/L yeast nitrogen base without amino acids, 5 g/L ammonium sulfate, 20 g/L glucose, added with aminoacids if required) was used to perform functional assays on transformed yeast cells, by monitoring for 3 days the optical density at 600 nm (OD_600_) of yeast cell cultures growing at 28 °C in either SD medium, or SD added with 2 mM CuSO_4_. When required, glucose in synthetic medium was replaced by 20–40 g/L of galactose (SG). The *E. coli* cells were grown in Luria-Bertani (LB; Bacto tryptone 10 g/L; Yeast extract 5 g/L; and NaCl 5 g/L) at 37 °C with 0.1 mg/mL ampicillin if required. Media components and chemicals, as reagents for auxotrophic requirements and antibiotics, were obtained from Difco (Thermo Fischer Scientific, Carlsbad, CA, USA), Sigma-Aldrich (Saint Louis, MO, USA) and Jena Bioscience GmbH (Malchin, Germany).

### Plasmids construction

**Fig. 1:**
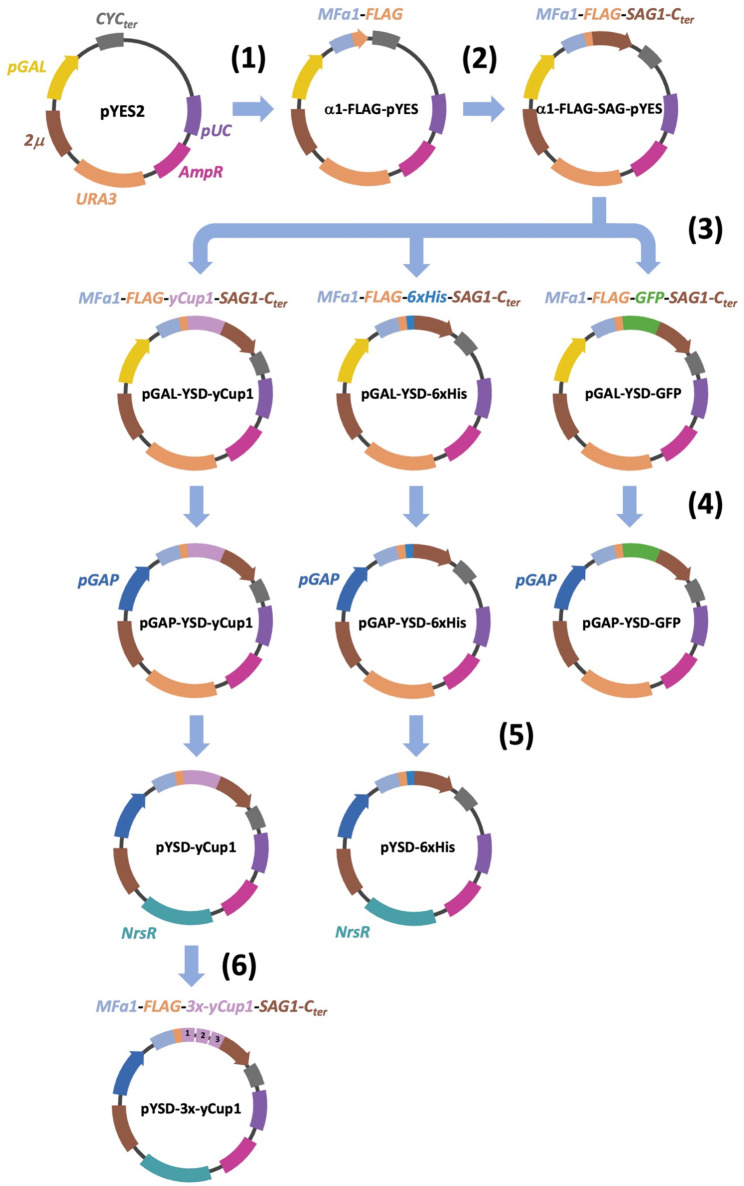
Yeast Surface Display (YSD) plasmids construction and maps. Yeast multicopy plasmid pYES2 was sequentially modified by the insertion of the MFα1 sequence fused to the FLAG tag (**1**), followed by the cloning of the cell wall anchor sequence (SAG1-C_ter_) (**2**). In the resulting vector, between MFα1-FLAG and SAG1-C_ter_ was (in-frame) cloned the coding sequence of either yeast metallothionein (yCup1), GFP protein, or exa-Histidine peptide (6xHis), generating pGAL-YSD plasmids series (**3**). The substitution of the pGAL1 (inducible) with the pGAP (constitutive) promoter originated the pGAP-YSD plasmids, constitutively expressing the chimeric proteins in yeast cells (**4**). Then, the nutritional yeast recessive URA3 marker was substituted by the dominant NrsR marker, to use pYSD plasmids in natural strains (**5**). Two additional copies of yCup1 protein were cloned in the pYSD-yCup1 plasmid to generate the chimeric protein with 3 repeats of yCup1 fused as tandem (**6**). Plasmid functional elements and their modifications are indicated by colors. *2µ ori*: yeast replication; *pUC ori*: *E.coli* replication; *AmpR*: *E.coli* selection marker; *CYCter*: yeast transcription termination sequence. The name of each plasmid is reported, accordingly to Supplementary Table ST2. Image was created with BioRender.com

The Yeast Surface Display (YSD) plasmids used in this work are listed in **Table ST2** and detailed experimental procedures for their construction are described in **Supplementary Material**. The sequences of the primers used are reported in Table ST3. All plasmids were derived from the multicopy, galactose-inducible pYES2 vector (Clontech) by sequential cloning steps (see Fig. 1). Briefly, the α-factor secretion signal sequence (MFα1, aa 1–85, FLAG-tagged) was first inserted into the multi-cloning site by the InFusion-HD cloning kit, following the manufacturer’s instructions (Takara-bio, USA, Inc. (San Jose, CA, USA), Cat. 102518). Similarly, the sequence of the cell wall anchor domain (SAG1, aa 330–650) was inserted downstream to MFα1. The InFusion kit was then used to in-frame cloning (between MFα1 and SAG1) of the coding sequence of different polypeptides (yCup1, GFP, exa-Histidine), generating the pGAL-YSD-yCup1, pGAL-YSD-GFP, and pGAL-YSD-6xHis plasmids, respectively. Thereafter, the galactose-inducible *GAL1* promoter was replaced by the promoter sequence of the constitutive *S.cerevisiae TDH3* gene (encoding isoform 3 of Glyceraldehyde-3-Phosphate Dehydrogenase (GAPDH)), resulting in the pGAP-YSD-GFP, pGAP-YSD-yCup1, and pGAP-YSD-6xHis plasmids. In the last two plasmids, the *URA3* marker was replaced by the *NatR* marker, conferring resistence to Nourseothricin antibiotic to yeast cells, generating the pYSD-yCup1 and pYSD-6xHis plasmids. The pYSD-3x-yCup1 plasmid expressing 3 repeats of the yCup1 protein fused in tandem was generated by the insertion of 2 additional copies of the yCup1 coding sequence into the pYSD-yCup1 plasmid, as detailed in **Supplementary Material**. The plasmid for CRISPR/Cas9 yeast genome modification in the *ADE2* locus (gADE-pMEL13-Cas9) was generated by the InFusion system as already described [34]. All recombinant plasmids were extensively controlled by DNA restriction analysis and Sanger sequencing. The resulting plasmid maps are shown in Fig. 1, and are available upon request.

### Yeast manipulation

*S. cerevisiae* cells were chemically transformed by the lithium acetate method [35, 36], and recombinant clones were selected and isolated incubating yeast transformed cells for 3 days at 28 °C on solid medium plates of either synthetic SD (lacking Uracil), or complete YPD medium supplemented with antibiotics (Nourseothricin or G418 Geneticin). CRISPR/Cas9 genetic modifications were performed as described previously [34], and yeast cells were cotransformed with 1 µg of guide gRNA/Cas9-expressing plasmid (gADE2-pMEL13-Cas9, targeting the *ADE2* locus) and 3 µg of donor dsDNA fragment, obtained by PCR amplification with the specific primers (**Table ST3**) of plasmids carrying either YSD-yCup1, or YSD-3x-yCup1 sequence. The genetic modifications were confirmed in the recombinant clones by site-specific PCR assays, using genomic DNA as template and the appropriate diagnostic primers (dgADE2-For and Rev). Procedure details are provided in the **Supplementary Material**.

### Yeast protein extraction and western blot analysis

Total proteins were extracted from the yeast cells following the trichloroacetic acid (TCA) method [37], with minimal variations (see **Supplementary Material**). For Western blot assays, 10–20 µg of total proteins were loaded in 10% acrylamide-N, N’-methylenebisacrylamide (37.5:1 (w/w) gel, and separated by SDS-polyacrylamide gel electrophoresis (SDS-PAGE). Proteins were then transferred on Polyvinylidene Difluoride (PVDF) membrane (Millipore Co., Boston, MA) using the TransBlot^®^ Turbo™ Transfer System (Bio-Rad Laboratories), and incubated (60 min, RT) in blocking solution (5% (w/v) nonfat dry milk (Bio-Rad Laboratories) in TRIS-buffered saline (TBS) supplemented with 0.1% (w/v) Tween-20 (TBS-T)). Membranes were probed (60 min, RT) with either anti-FLAG (1:2000, Sigma-Aldrich, cat. F7425), or anti- GAPDH (1:10000, Abcam, Cambridge, UK, cat. AB9485) rabbit primary antibodies, diluted in TBS-T added with 1% (w/v) bovine serum albumin (BSA). After tree washes with TBS-T, the membranes were incubated with the secondary horseradish peroxidase-conjugated anti-(rabbit)IgG antibody (1:6000, Sigma-Aldrich, cat. A6154) diluted in TBS-T supplemented with 1% (w/v) bovine serum albumin (BSA) (60 min, RT). After double TBS-T washes, immunoreactive bands were visualized by the enhanced chemiluminescence reagent kit (EMD Millipore, Darmstadt, Germany), and finally digitalized by the UVItec imaging system (Eppendorf, Hamburg, Germany). Protein loading was checked by either GAPDH detection, or staining with Coomassie brilliant blue (Sigma-Aldrich).

### Immunocytochemistry and confocal microscopy

Yeast cells grown overnight in YPD medium were fixed in 3,7% formaldehyde (15 min, RT) and incubated with DAPI (Sigma-Aldrich, 2 µg/mL final concentration) to stain nuclear DNA. The cells were then washed twice with phosphate-buffered saline (PBS) buffer and incubated in PBS supplemented with 1% (w/v) bovine serum albumin (BSA) (60 min, RT). Anti-FLAG primary antibody was added (1:200 in PBS-BSA 1%) and incubated for 60 min (RT). After three washes with PBS, the cells were probed for 60 min (RT) with the secondary Alexa Fluor 555-conjugated anti-(rabbit) IgG antibody (Invitrogen, cat. A21428) (1:200 in 1% PBS-BSA), washed with PBS and finally resuspended in 1 M sorbitol. Cell suspensions were dropped onto a slide (Superfrost, BDH) and covered with a coverslip. Yeast cells were observed with a Leica SP5 confocal microscope using 63X HCX PL APO (NA 1.4) oil-immersion objectives. Laser excitation line, power intensity, and emission range were chosen according to the fluorophore in different samples to minimize bleed-through. During acquisition, parameters for laser intensity and photomultiplier gain were kept constant. Imaging was performed at 1024 × 1024 pixels, with a 200 Hz acquisition rate. Images were processed using Fiji/ImageJ software.

### Metal adsorption assay and ICP-OES analysis

Metal adsorption by yeast cells was performed by adapting previously described experimental protocols [4, 5, 38–40]. Briefly, yeast cells grown overnight in liquid medium were harvested and washed twice in PBS (pH = 7). Then, the cells were resuspended and incubated for 2 h at 28 °C in PBS supplemented with either 10 µM CuSO_4_, or NiSO_4_, to a final concentration of 5 × 10^8^ cells/mL (binding phase). Thereafter, the cells were sedimented, washed twice with PBS, and finally incubated in 1 mL of 5 mM EDTA with rotation at 4 °C for 30 min to dissociate the metal ions from the cells (recovery phase). Cells were separated by centrifugation and supernatant (1 mL) of each sample was supplemented with 5 µl of 68% nitric acid for stabilization. Heavy metals concentration measurments were performed by Inductively Coupled Plasma– Optical Emission Spectroscopy (ICP-OES, Agilent Tecnologies 5110, Santa Clara, California, United States). For copper analysis, the samples were diluted 4 times with 5% nitric acid and filtered (0.45 μm pore). The samples were individually analyzed three times for 30 s at λ = 324,754 nm for copper and λ = 231,604 nm for nickel detection, and the means were considered as the correct values for the copper content, which were expressed in ppm. Data were analysed and reported in graphs as nmoles of ions detected for nickel, or nmoles normalized to milligrams of Dry Cell Weight (DCW) per liter, considering the correlation 1 OD_600_ = 0.62 gDCW/L [41].

### Statistical analysis

Data were analyzed using Prism 8 (GraphPad Software) and Microsoft Excel (Microsoft Corporation) software. Data are reported as the mean ± Standard Error of the Mean (SEM). The number of biological replicates (n) reported in the figure legends indicates the number of experiments performed on different days. Depending on the experiment, the statistical analysis was carried out as indicated in each figure caption. A p-value < 0.05 was considered to indicate statistical significance (∗*p* < 0.05, ∗∗*p* < 0.01).

## Results and discussion

### Construction of the yeast plasmids for protein expression on outer cell surface

To establish the YSD system, we selected three different polypeptides of different size as model examples for expression on the yeast cell wall: the cytosolic metallothionein of *S.cerevisiae* (yCup1p), the heterologous fluorescent protein mNeonGreen [42], and the small peptide exa-Histidine (6xHis). Initially, we constructed pYES2-derived plasmids to express these chimeric polypeptides, which were N-terminally tagged with a FLAG epitope, under the control of the galactose-inducible *GAL1* promoter. We sequentially inserted into the multicopy pYES2 plasmid the pre-pro-peptide sequence of yeast α-factor (MFα1), followed by the FLAG epitope, the respective coding sequences for the protein/peptide (yCup1, GFP, or 6xHis), and the signal for cell wall anchoring (SAG1-C_ter_), thereby generating the corresponding pGAL-YSD-(yCup1/GFP/6xHis) plasmids (Fig. 1).

Yeast cells of the CENPK laboratory strain were first transformed with the pGAL-YSD plasmids, carrying either yCup1 or GFP chimeric transgenes. After growing the cells in an inducing medium containing galactose, total proteins were extracted. Western blot analysis confirmed that both yCup1 and GFP fusion proteins were successfully expressed in yeast cells, as evidenced by the specific bands of the expected sizes detected by the anti-FLAG antibody. The data showed that protein expression occurred in a time-dependent fashion, with maximal levels observed after 18 h of incubation in 2% galactose medium (**Fig. S1a**). Consistently, when the galactose concentration was increased to 4%, maximal expression was achieved earlier, after 12 h of induction (**Fig. S1b**). The same observations on protein expression were also made for 6xHis fusion protein (data not shown). Importantly, no differences in growth rates were observed between strains transformed with the plasmids and control cells (carrying the empty vector), indicating that the expression of chimeric proteins did not impair yeast viability or proliferation.

**Fig. 2:**
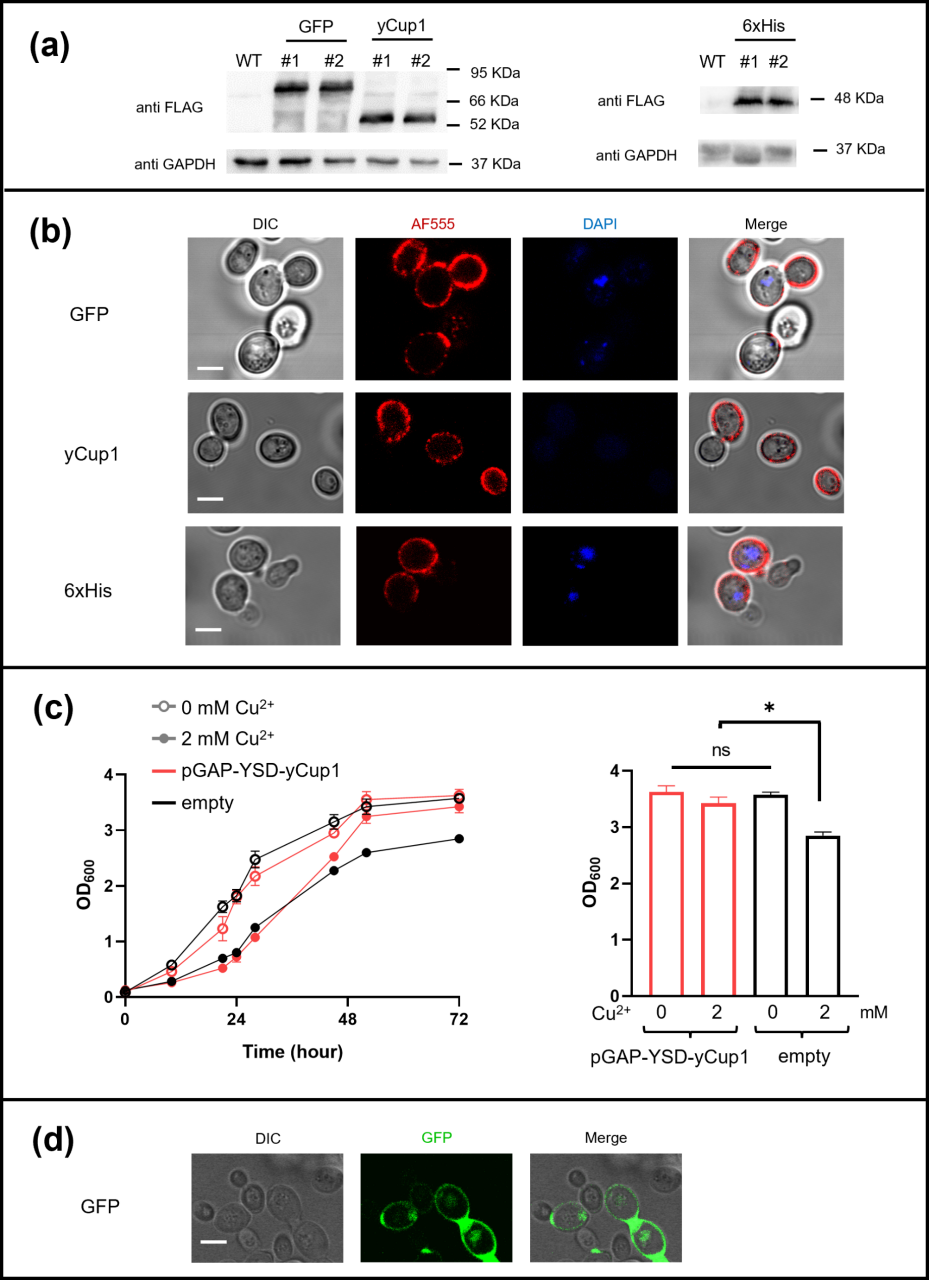
Analysis of the YSD plasmids in yeast laboratory strain. (**a**) Total proteins from CENPK laboratory cells (two independent clones, #1–2), carrying either pGAP-YSD-GFP, pGAP-YSD-yCup1, pGAP-YSD-6xHis plasmids, or the empty vector as control (WT), were subjected to Western blot assay using the anti-FLAG antibody and with anti-GAPDH antibody. Images are representative of three independent experiments. **(b)** CENPK cells transformed as in (a), were analyzed by immunofluorescence microscopy to visualize the localization of the fusion proteins. For each strain, micrographs are reported of the differential interference contrast (DIC), the immunofluorescence signal (AF555), the nuclear DNA staining (DAPI), and the merged image. Images are representative of three biological replicates for each yeast strain. Scale bar: 5 μm. **(c)** Growth curves of yeast CENPK cells transformed with either pGAP-YSD-yCup1 (*red*) or empty (*black*) plasmids, incubated in SD medium (*empty dots*), or SD added with 2mM of CuSO_4_ (*filled dots*). Growth rate was monitored for 72 h by OD_600_ measurement (*left panel*). The OD_600_ final values (72 h) are also reported as graph (*right panel*). Each column is the mean ± SEM of 4 independent experiments (*n* = 4, *p < 0,05, ONE WAY Anova with Kruskal-Wallis test was applied for statistical analysis). **(d)** Living CENPK cells carrying the pYSD-GFP plasmid were directly observed by confocal microscopy. Green fluorescence signal (GFP), differential interference contrast (DIC) micrograph, and merge are reported. Image is representative of three biological replicates; scale bar = 5 μm

Subsequently, the *GAL1* promoter of the pYES2-derived vectors was replaced with the constitutive GAP (glyceraldehyde-3-phosphate dehydrogenase) promoter to enable continuous expression of the chimeric proteins (see Fig. 1). The resulting vectors were used to transform CENPK cells, and protein expression was confirmed as described above. Total proteins from yeast cells transformed with either pGAP-YSD-GFP, pGAP-YSD-yCup1, or pGAP-YSD-6xHis plasmids, as well as from control cells (transformed with an empty vector), were subjected to Western blot analysis using an anti-FLAG antibody to detect FLAG-tagged chimeric proteins. Anti-GAPDH antibody was used as a loading control. The results (Fig. 2a) indicated that the plasmids were able to drive the constitutive expression of both orthologous and heterologous chimeric proteins in CENPK yeast cells, similarly to the conditional (galactose-inducible) plasmids described above.

To ensure that the chimeric proteins were correctly localized on the yeast cell wall, we performed immunofluorescence analysis to examine the distribution of yCup1-, GFP-, and 6xHis-fusion proteins in CENPK cells transformed with the constitutive plasmids. As observed in similar studies [4, 5], intact (non-permeabilized) cells were directly probed with the anti-FLAG antibody, and the FLAG-tagged fusion proteins were clearly localized on the yeast outer surface (Fig. 2b), demonstrating that the constitutive plasmids efficiently directed the expression of chimeric proteins to the cell wall, thus strongly supporting their efficiency as molecular tools for the YSD system. Notably, we tested the plasmids in another laboratory strain (W303) and obtained comparable results for both protein expression and surface localization, further confirming the functionality of the plasmids across different genetic backgrounds (Fig. S2).

Given that prior studies [38, 43] have shown that surface-exposed functional metal-binding proteins can enhance yeast resistance to metal-induced cytotoxicity, we evaluated the functionality of the copper-binding yCup1 metallothionein expressed on the yeast cell wall via the pGAP-YSD-yCup1 plasmid. We compared the growth of transformed CENPK cells in copper-containing medium to control cells carrying the empty vector. Transformed cells were incubated in stationary phase in SD medium, then diluted to an OD_600_ = 0.1 in either SD medium alone, or SD medium supplemented with 2 mM CuSO_4_. Growth rates were monitored by measuring OD_600_ over 72 h. The results (Fig. 2c) showed that the presence of 2 mM copper significantly inhibited the growth of control cells, while cells expressing yCup1 on their surface exhibited substantial resistance to copper toxicity, behaving similarly to those grown in copper-free medium. This finding suggested that yCup1 maintained its biological activity when displayed on the cell surface. Furthermore, the functionality of surface-exposed proteins was corroborated by the detection of green fluorescence emitted by GFP expressed on the yeast cell wall by the pGAP-YSD-GFP plasmid. This fluorescence was directly observed by confocal microscopy in living cells, as shown in Fig. 2d.

In conclusion, our data demonstrated that both constitutive and conditional plasmids effectively drive the expression of orthologous and heterologous chimeric proteins in yeast cells. These proteins are properly targeted to the outer cell surface (i.e., cell wall) and retain their functional properties, highlighting the potential for using these systems in diverse applications.

### Analysis of the yeast surface display (YSD) plasmids in natural strains

To assess protein expression driven by YSD plasmids in natural yeast strains, the nutritional (recessive) marker (*URA3*) of the pYES2-derived vectors was replaced with the *NatR* dominant marker, which confers resistance to the antibiotic Nourseothricin in yeast cells (see Fig. 1). Considering their possible use in natural strains, in particular as metal-binding tools, only vectors constitutively expressing the yCup1 and 6xHis proteins were generated (i.e., pYSD-yCup1 and pYSD-6xHis), and the pYSD-yCup1 plasmid was first used to transform several natural *S. cerevisiae* strains (IB1–IB8) from the private collection of Italiana Biotecnologie S.r.l., commonly employed in winemaking for must fermentation.

**Fig. 3:**
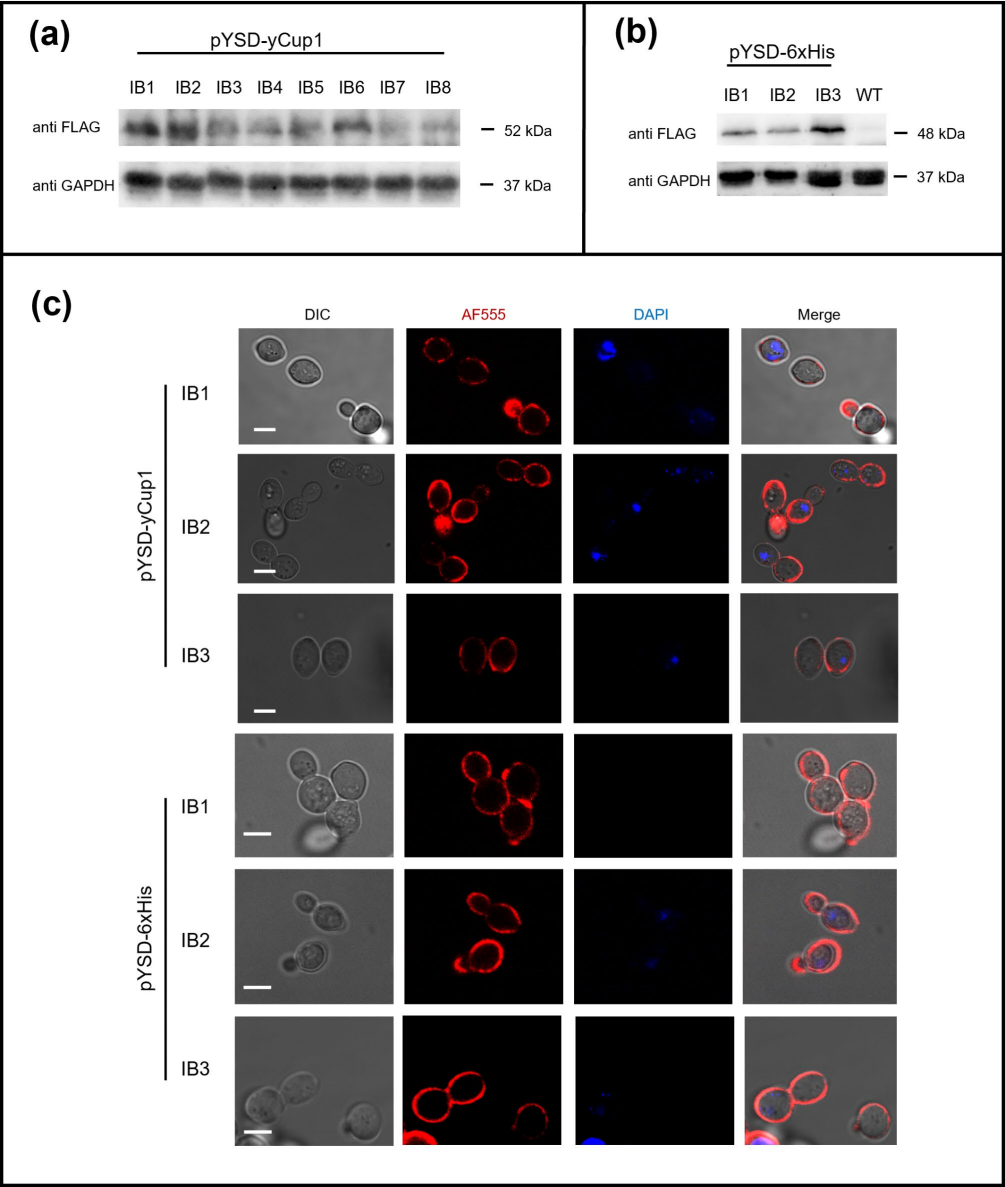
Analysis of the YSD plasmids in natural yeast strains. (**a**) Total proteins from 8 natural yeast strains (IB1-IB8), carrying the pYSD-yCup1 plasmid, were analyzed by Western blot as indicated in Fig. 2. (**b**) Western blot assay performed for the IB1-IB3 natural strains transformed with pYSD-6xHis. The GAPDH protein was used as loading control. Images are representative of three independent experiments. **(c)** Yeast IB1-IB3 strains carrying either pYSD-yCup1, or pYSD-6xHis plasmids, were analyzed by immunofluorescence microscopy to visualize the localization of fusion proteins. For each strain, micrographs are reported of the differential interference contrast (DIC), the immunofluorescence signal (AF555), the nuclear DNA staining (DAPI), and the merged image. Images are representative of three biological replicates for each yeast strain. Scale bar = 5 μm

Total proteins were extracted from each transformed yeast strain and subjected to Western blot analysis, as previously described. The experimental results (Fig. 3a) demonstrated that all yeast strains expressed the FLAG-tagged yCup1 fusion protein, although expression levels varied between strains, as indicated by the differing signal intensities detected by the FLAG antibody. As mentioned, this variability could reflect strain-specific features, such as differences in cell wall composition/remodeling or in the efficiency of the secretory pathway.

For subsequent experiments, we selected three natural strains (IB1, IB2, and IB3) that expressed comparable levels of the fusion protein but exhibited distinct phenotypic traits, such as varying levels of resistance to copper toxicity (IB1 > IB2 > > IB3, **Fig. S3**). These strains were then transformed with the pYSD-6xHis plasmid and analyzed in the same manner to evaluate whether the exa-Histidine fusion protein was also expressed and properly localized in the cell wall. Western blot assays (Fig. 3b) and immunofluorescence analysis (Fig. 3c) confirmed that both the yCup1 and the exa-Histidine chimeric proteins were successfully expressed and distributed on the cell surface in the natural strains. Importantly, the results demonstrated that protein expression driven by the pYSD plasmids was independent of the yeast genetic background, as indicated by similar performance across multiple strains from both laboratory and natural sources. Moreover, very similar results were observed when plasmids were used to transform *S.cerevisiae* strains isolated for non-oenological applications, as production of beer, bread, or whisky (*data not shown*). Overall, data indicated the plasmids generated here may represent universal tools to perform YSD in the yeast strains belonging to *S.cerevisiae* species isolated from natural environment. Notably, some DNA elements used in plasmid backbone (e.g., promoters, terminators, markers), as well as protein domains (e.g., secretion signal sequence), have been already successfully employed in different yeast species such as *Pichia pastoris*,* Yarrowia lipolytica* or *Hansenula polymorpha*, suggesting the pYSD plasmids could be functional also in such species, that is currently under investigation. Nevertheless, the YSD strategy may be easily adapted by the replacement of specific portions of the chimeric protein (e.g., cell wall anchoring) with similar signal sequences identified in the target species, which are commonly used for various biotechnological purposes (e.g. bioethanol production), and could be in turn implemented via YSD system.

### Metal binding by yeast cells expressing the chimeric proteins on the surface

Since the ability of the yCup1 metallothionein to bind copper ions (Cu²⁺) had been established [44], and the affinity of the exa-Histidine peptide for nickel ions (Ni²⁺) was well-documented [38, 45], we conducted Inductively Coupled Plasma– Optical Emission Spectroscopy (ICP-OES) assays to evaluate the binding of copper and nickel ions to yeast cells expressing the yCup1 or exa-Histidine fusion protein, respectively, in comparison to control (non-transformed) cells. As described above (Sect. 2.6), we adapted experimental protocols reported in similar studies on yeast cells, with minimal modifications [4, 5, 38–40]. Briefly, yeast cells (5 × 108) were incubated in metal-supplemented medium to allow for metal binding, followed by chemical treatment with EDTA to dissociate the metal ions (recovery). After yeast sedimentation, metal concentrations in the supernatants were measured by ICP-OES. In this study, we analyzed three natural diploid strains (IB1, IB2, and IB3) and compared them to the diploid W303 strain, considered as a reference for laboratory *S. cerevisiae* strains.

**Fig. 4:**
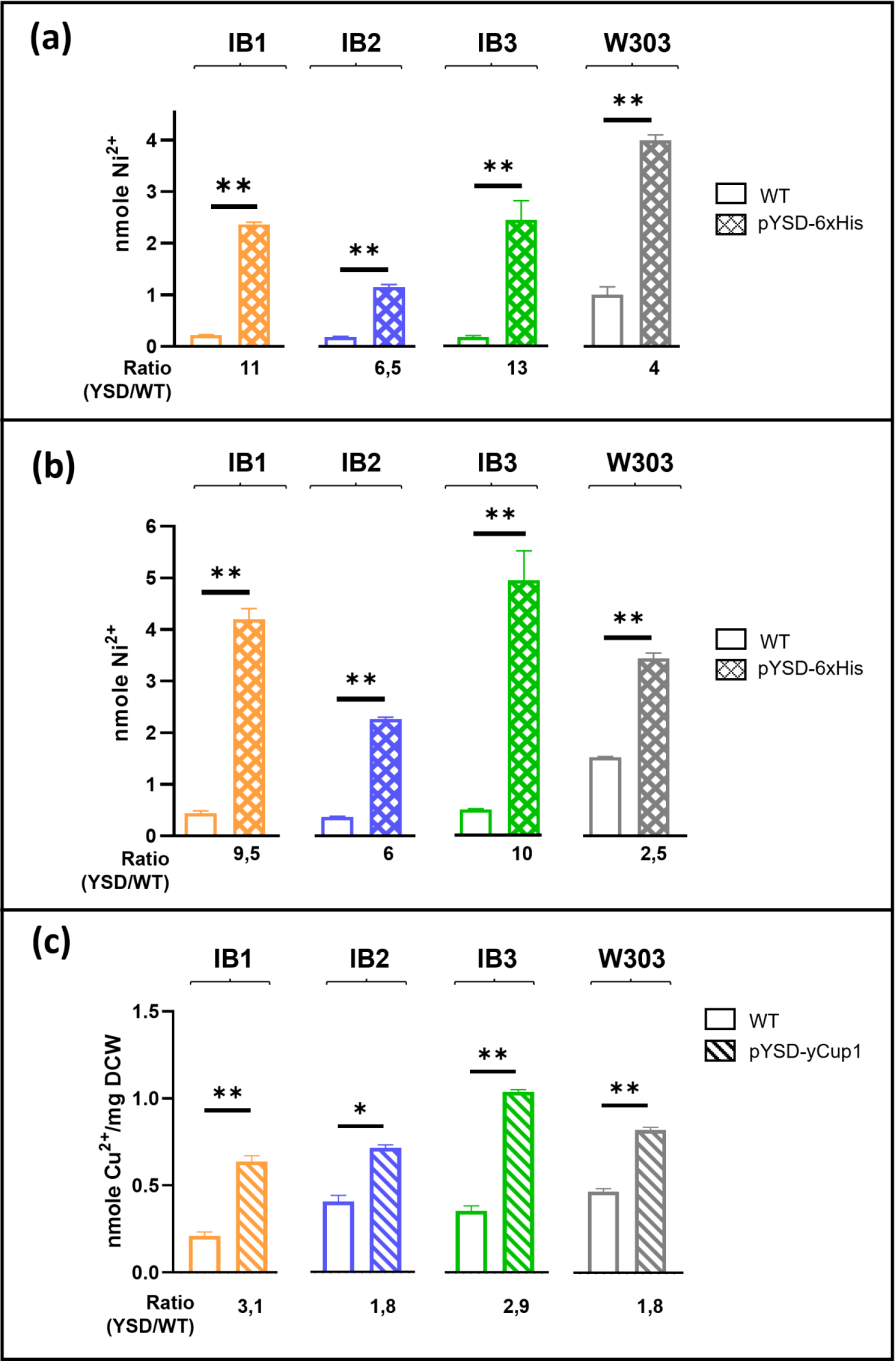
Metal binding by yeast strains expressing chimeric proteins on the outer surface. **(a)** 5 × 10^8^ yeast cells of each indicated strain (parental (WT) or carrying the pYSD-6xHis plasmid), were used for Ni^2+^ binding experiments as described, and ICP-EOS assays were performed to quantify the nmoles of metal ions, reported in the graphs. The ratio between values of modified and parental strains (YSD/WT) is indicated for each strain. **(b)** Similar experiments were performed using double (10^9^) amount of yeast cells. **(c)** As in (a), 5 × 10^8^ yeast cells, either parental (WT) or carrying the pYSD-yCup1 plasmid, were used for Cu^2+^ binding experiments, and Cu^2+^ ions quantification data are presented as nmoles of Cu^2+^ normalized to mg of CDW. In all panels, each column represents the mean ± SEM of 5 independent experiments (*n* = 5; **p < 0,01, Mann Whitney T-test was applied for statistical analysis)

Experimental data demonstrated that expressing the exa-Histidine sequence on the cell surface significantly increased nickel ion binding in all yeast strains compared to their parental strains (Fig. 4a). We calculated the protein-dependent increase as the ratio between the Ni²⁺ levels detected in transformed (YSD) and non-transformed (WT) cells. This increase was more than tenfold in two natural strains (IB1 and IB3). Further data supported that nickel binding occurred in a dose-dependent manner, as doubling the number of yeast cells (1 × 10^9^) resulted in a proportional increase in the recovery of metal ions across all yeast strains (Fig. 4b). Interestingly, the basal level of metal binding appeared to be strain-specific, as evidenced by the differences in nickel levels bound by the parental strains.

Additional ICP-OES data revealed that expressing the yCup1 protein on the cell surface also significantly increased copper ion binding across all yeast strains compared to their parental cells (Fig. 4c). Again, the intrinsic ability of the yeast strains to bind copper varied, as indicated by the copper levels in the parental strains. However, these findings demonstrate that the metal-binding capacity of all strains displaying the surface-bound yCup1 protein was significantly enhanced, although the extent of this enhancement varied. Importantly, the copper levels we detected were consistent with previously reported measurements [38, 39].

Interestingly, copper binding by the yeast cells seemed to be unrelated to their sensitivity to metal toxicity, as similar copper-binding performances were observed in strains with very different resistance to copper (e.g., IB2 and IB3, see **Fig. S3**). Instead, copper binding might be influenced by the intrinsic hydrogen sulfide (H₂S)-producing capacity of the strains, as recently suggested [46]. Our previous observations indicated that, compared to the IB1 strain, the IB3 strain is a high H₂S producer, which may explain its greater ability to bind copper ions.

### Genetic modifications of natural yeast strains by CRISPR/Cas9 system

**Fig. 5:**
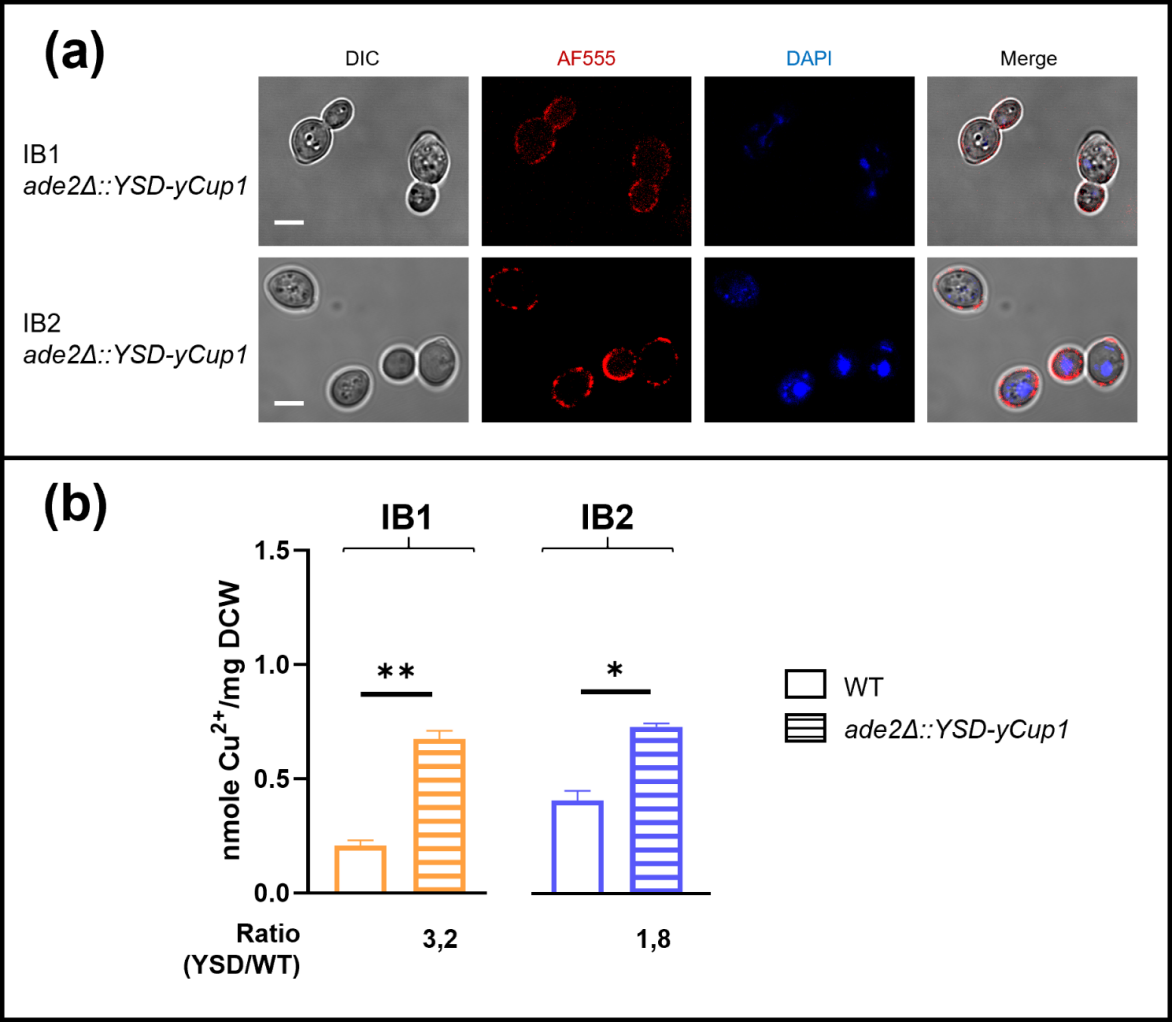
Analysis of genetically modified yeast cells. **(a)** Yeast IB1- and IB2-derivative strains, carrying one chromosomal copy of the YSD-yCup1 transgene replacing *ADE2* gene, were analyzed by immunofluorescence microscopy to visualize the localization of the fusion protein. For each strain, micrographs are reported of the differential interference contrast (DIC), the immunofluorescence signal (AF555), the nuclear DNA staining (DAPI), and the merged image. Images are representative of three biological replicates for each yeast strain. Scale bar: 5 μm. **(b)** 5 × 10^8^ yeast cells of the IB1 and IB2 strains, either unmodified (WT), or carrying one chromosomal copy of the yCup1 chimeric transgene (*ade2D::YSD-yCup1*), were checked for Cu^2+^ binding by ICP-EOS and quantification data are reported in the graphs (as nmoles of Cu^2+^ / mg CDW). The ratio between values of modified and parental strains (YSD/WT) is indicated for each strain. Each column represents the mean ± SEM of 5 independent experiments (*n* = 5; *p < 0,05, **p < 0,01, Mann Whitney T-test was applied for statistical analysis)

In the yeast cell, the efficiency of ectopic expression on the outer surface primarily depends on the cellular copy number of the plasmids carrying the transgene, often leading to considerable clonal variability in protein expression levels. To overcome such limitation, we generated yeast strains that stably integrated into the genome the DNA sequence required to constitutively express the chimeric protein on the cell surface. Notably, two natural yeast strains (IB1 and IB2) were modified by inserting the complete YSD-yCup1 expression cassette into the *ADE2* locus using the CRISPR/Cas9 system (see Sect. 2.3). We verified the genetic modifications using locus-specific PCR assays (**Fig. S4a**) and confirmed the expression and surface localization of the yCup1 fusion protein through immunofluorescence assays (Fig. 5a). Subsequently, we evaluated the copper-binding capability of the genetically modified strains using ICP-OES analysis, as described. The genomic integration of the YSD-yCup1 transgene significantly enhanced copper binding in both IB1 and IB2 strains compared to their parental counterparts (Fig. 5b). Notably, the improvement in metal-binding properties was comparable to that achieved with the multicopy pYSD-yCup1 plasmid, indicating that a single chromosomal copy of the YSD-yCup1 transgene may be sufficient to maximize the metal-binding capacity of the modified yeast cells.

**Fig. 6:**
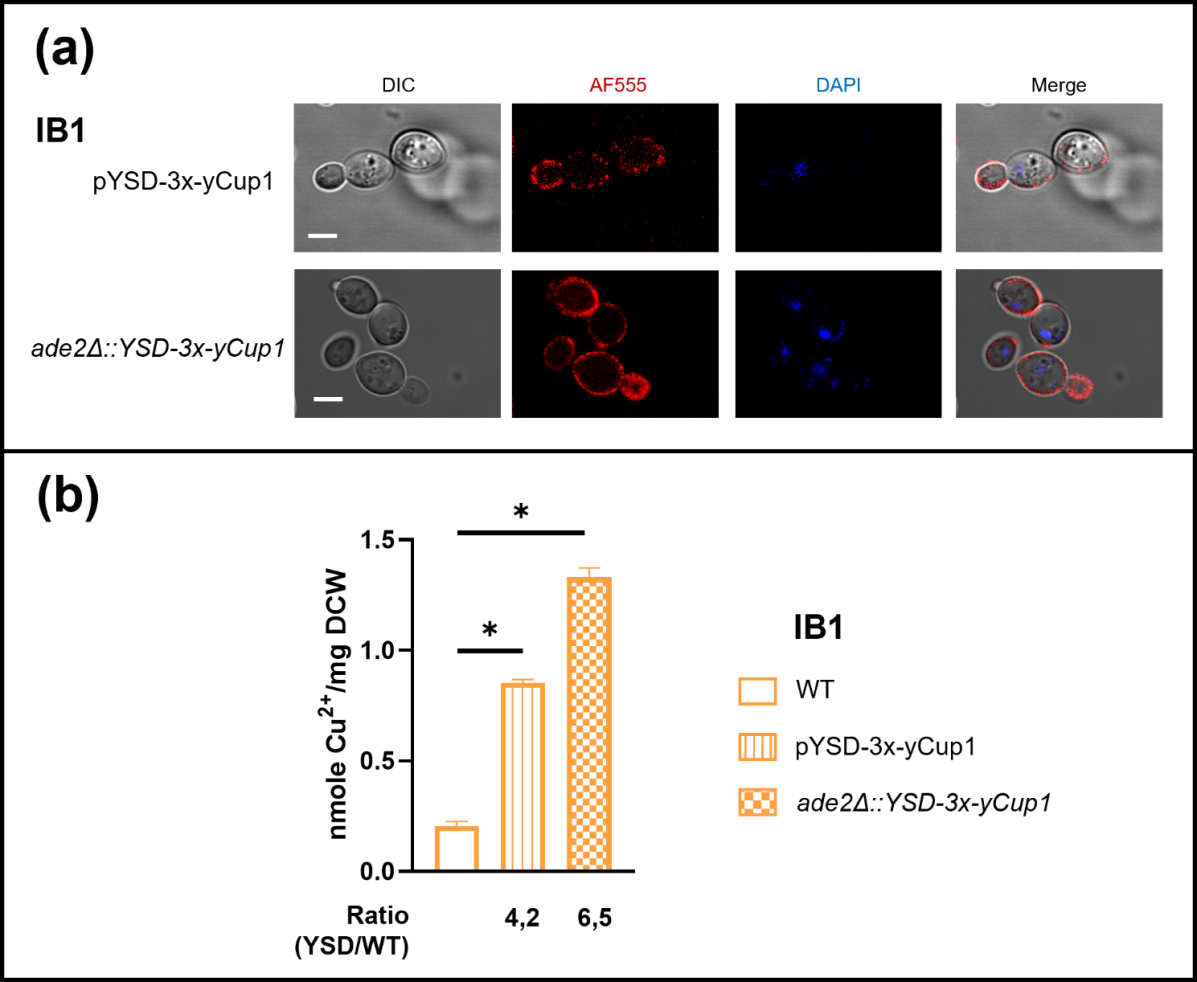
Analysis of IB1 yeast cells expressing multiple binding units **(a)** Yeast IB1 cells either transformed with the pYSD-3x-yCup1 plasmid, or carrying one chromosomal copy of the *YSD-3x-yCup1* transgene in the *ADE2* locus, were analyzed by immunofluorescence microscopy to visualize the localization of the fusion protein. For each strain, micrographs are reported of the differential interference contrast (DIC), the immunofluorescence signal (AF555), the nuclear DNA staining (DAPI), and the merged image. Images are representative of three biological replicates for each yeast strain. Scale bar: 5 μm; **(b)** 5 × 10^8^ yeast cells of either unmodified IB1 strain (WT), cells expressing triple-tandem yCup1 chimeric protein by multicopy plasmid (pYSD-3x-Cup1), or by single chromosomal copy of the transgene (*ade2D::YSD-3x-yCup1*), and Cu^2+^ binding was evaluated by ICP-OES, as previously described. Each column represents the mean ± SEM of 4 independent experiments (*n* = 4; *p < 0,05, Mann Whitney T-test was applied for statistical analysis)

As previously reported [5, 40], expressing multiple metal-binding proteins as tandem repeats can further enhance metal-binding properties. Therefore, we generated the pYSD-3x-yCup1 plasmid for constitutive expression of a fusion protein containing three metallothionein repeats in tandem, that was used to transform the natural IB1 yeast strain. We assessed the fusion protein’s expression and confirmed its localization on the cell wall via immunofluorescence assays (Fig. 6a, **upper panel**). Additionally, we stably integrated the YSD-3x-yCup1 transgene into the IB1 genome by replacing the *ADE2* gene. Accuracy of the genetic modification was verified by locus-specific PCR (**Fig. S4b**), and expression and localization of the chimeric protein were confirmed by immunofluorescence analysis (Fig. 6a, **bottom panel**). The copper-binding ability of yeast cells expressing the 3-tandem yCup1 protein was evaluated using ICP-OES. The data showed that strains expressing the 3x-yCup1 protein, whether transformed with the multicopy plasmid or genomically integrated, exhibited significantly increased copper-binding properties compared to the parental IB1 strain (Fig. 6b). Specifically, copper binding was approximately 4 times higher in plasmid-transformed cells and 6 times higher in strains carrying a single chromosomal copy of the YSD-3x-yCup1 transgene compared to unmodified cells.

Overall, the experimental evidence supports that the fusion proteins encoded by the YSD cassette are efficiently expressed and correctly targeted to the cell surface while retaining their biological activity. Data thus point to the role of our plasmids as starting YSD platform to express any protein-of-interest in natural strains by one single vector. This study also provides the first experimental confirmation of enhanced metal-binding properties in natural yeast strains using the YSD strategy, a phenomenon previously described only in laboratory strains of *S. cerevisiae*. Future research should address several unresolved specific questions, including metal affinity, binding specificity, and the performance of YSD-armed strains in complex environments such as contaminated wastewater, where multiple metal ions at varying concentrations may compete for binding sites. Additionally, exploring other formats such as dead cells or peels could be beneficial. As reported [47], dead cells might offer advantages in terms of metal binding efficiency, pH variation tolerance, and resistance to ion concentration changes (no toxic effects), depending on their previous physical or chemical treatments. Importantly, the integration of YSD cassette into yeast genome provides a significant advantage, as these cells can be propagated in standard media without antibiotic requirements, reducing the application of large quantities of such expensive molecules, but also preventing its prolonged use, which may lead to the development of antibiotic resistance, finally making such engineered strains ideal for large-scale biomass production at industrial level. The ability to bind copper ions of the engineered yeasts expressing the YSD-yCup1 or YSD-3x-yCup1 transgenes could be of special interest to treat natural matrices enriched in this heavy metal, as it occurs in winemaking when the presence of high copper concentration in grape must could affect the fermentative process. However, as YSD-modified strains consist of transgenic organisms, their use is currently not allowed in some countries (e.g., EU), but admitted in other (e.g., USA), where the application of such GMO strains could be helpful to wine industries.

## Conclusions

The main goal of this work was to develop novel molecular tools that support the YSD system in natural strains of *S. cerevisiae*. Our experimental data demonstrated that our plasmids effectively drive the expression of chimeric proteins on the outer cell surface across multiple yeast strains, making them suitable tools for the modification of nearly all *S. cerevisiae* strains isolated from nature. In the future, such engineered yeasts could unveil promising/suitable features as biological platform for multiple applications, primary biocatalysis.

Importantly, our findings confirm that the metal adsorption properties of natural yeast strains can be significantly improved by displaying metal-binding proteins on the cell surface using the YSD system we developed, based on a single, selectable plasmid. This may suggest a potential application for bioremediation, extending the use of yeast strains previously considered for other purposes (e.g., oenology), but not for metal removal. Moreover, these strains represent only a small fraction of the multitude of *S. cerevisiae* strains present in nature yet to be isolated. Some of these strains may possess even more advantageous properties for metal decontamination through eco-friendly strategies, such as enhanced adsorption capacity and stress resistance.

## Electronic supplementary material

Below is the link to the electronic supplementary material.

Supplementary Material 1

## Data Availability

No datasets were generated or analysed during the current study.
