# Supplementary material for: Functional improvement of natural Saccharomyces cerevisiae yeast strains by cell surface molecular engineering
Source: Biol Direct. 2025 Feb 14;20:22. doi: 10.1186/s13062-025-00614-1 (PMC11829369; doi:10.1186/s13062-025-00614-1)
Supplement: Supplementary file 1 — Supplementary Material 1 [file 13062_2025_614_MOESM1_ESM.pdf]

Supplementary Material

"Functional improvement of natural *Saccharomyces cerevisiae* yeast strains by cell surface molecular engineering"

Sara Granuzzo, Monica Rossetto, Lucio Zennaro, Francesca Righetto, Paolo Antoniali, Raffaele Lopreiato*

Email: raffaele.lopreiato@unipd.it

This pdf file includes:

Supplementary Figures **S1** to **S4**

Description of the yeast strains used in this work

Additional experimental methods

Tables **ST1** to **ST3**

Supplementary References

Figures.

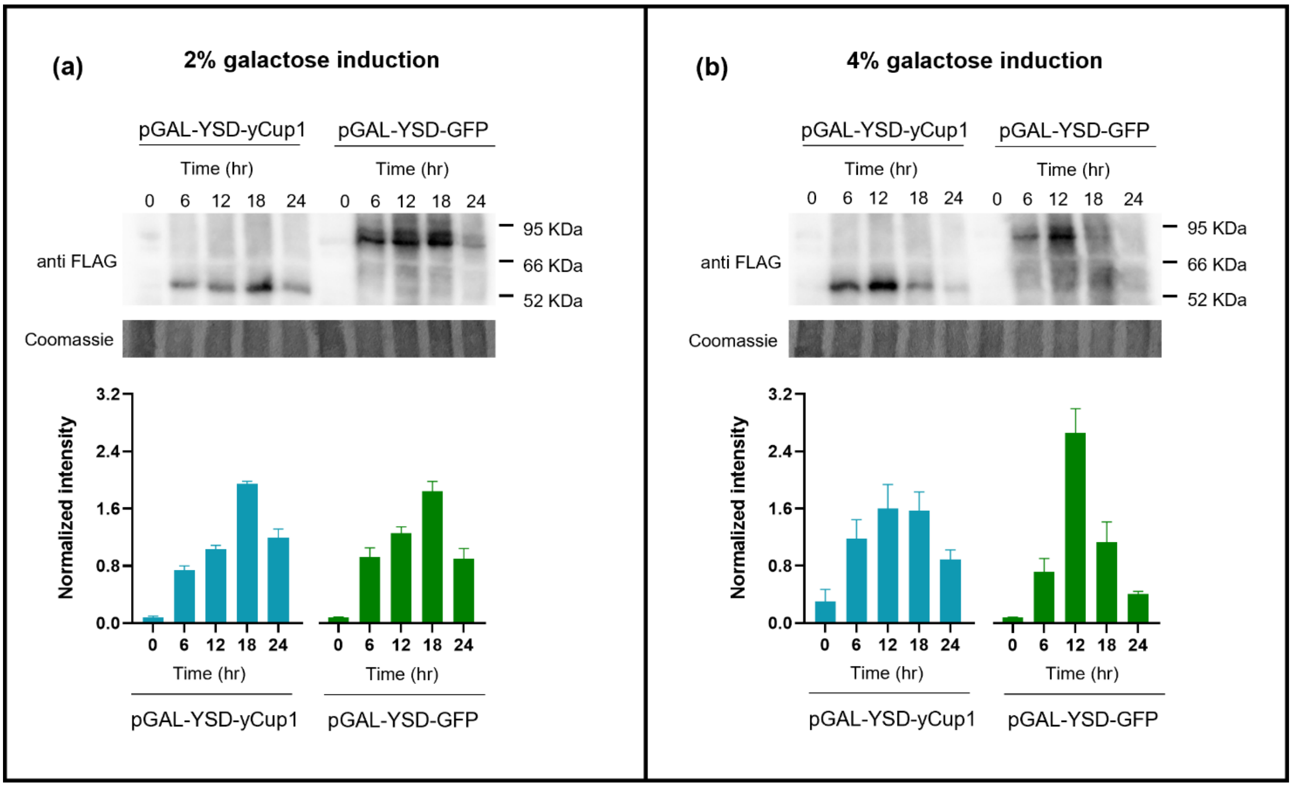

Figure S1. Kinetics of Yeast Surface Display protein expression in *S.cerevisiae* laboratory strains. Total proteins from CENPK yeast cells, carrying the indicated plasmid, were analyzed by Western Blot using anti-FLAG antibody to detect the expression of FLAG-tagged chimeric proteins over induction time. Cells were incubated for the indicated times in medium containing either galactose 2% (panel **a**) or 4% (panel **b**), total proteins were extracted and subjected to Western blot assay. Membranes were stained with Coomassie blue as loading control. Data quantifications are shown in bottom graphs (n=4).

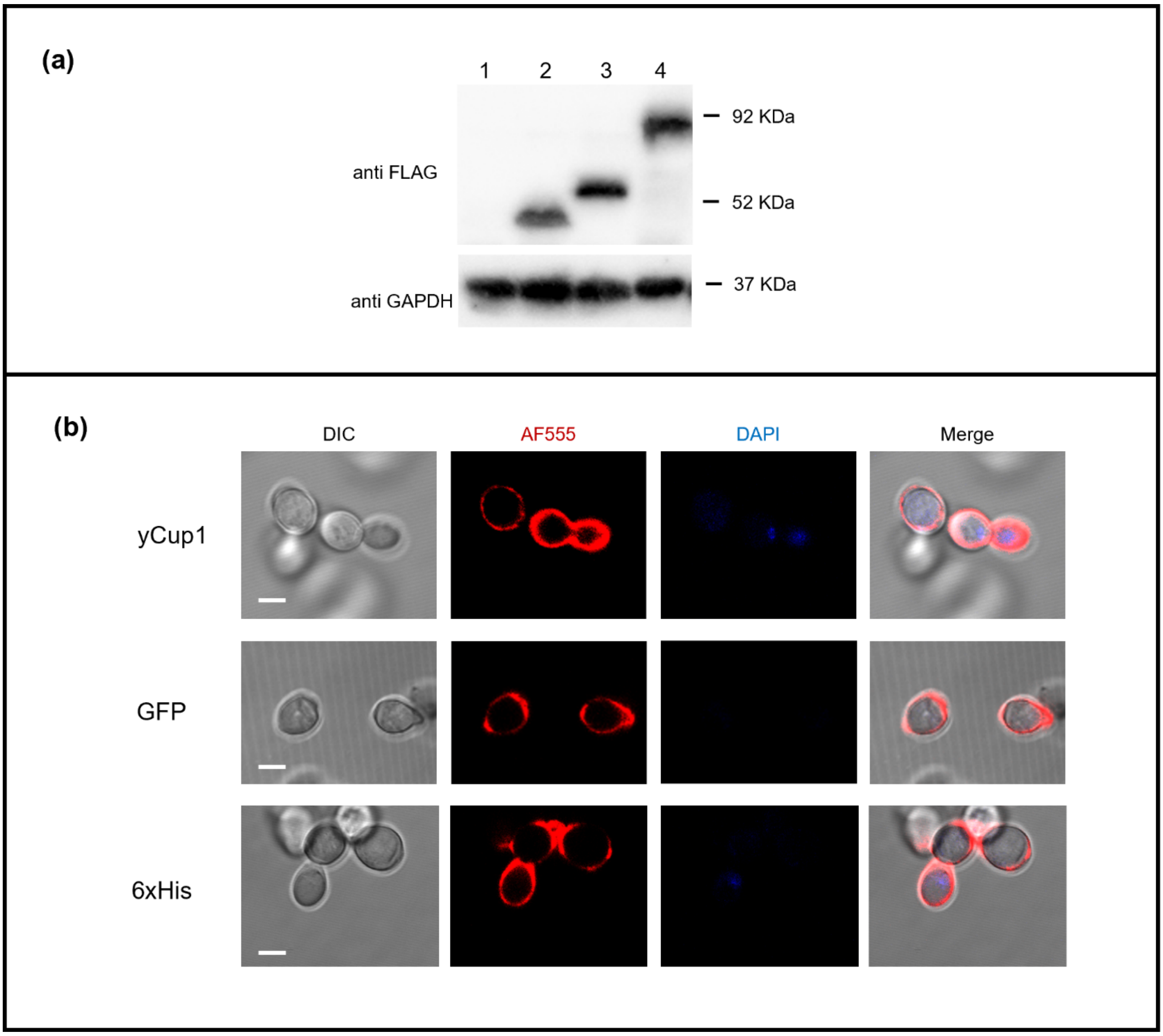

Figure S2. Analysis of the pGAP-YSD plasmids in yeast laboratory W303 strain. (a) Total proteins from W303 laboratory cells, transformed with either empty vector (lane 1), pGAP-YSD-6xHis (lane 2), pGAP-YSD-yCup1 (lane 3), pGAP-YSD-GFP (lane 4) plasmids, were subjected to Western blot assay using the anti-FLAG antibody to detect the expression of FLAG-tagged chimeric proteins. Membranes were also probed with anti-GAPDH antibody to verify proper protein loading. Images are representative of three independent experiments. **(b)** W303 cells carrying either pGAP-YSD-yCup1, pGAP-YSD-GFP, or pGAP-YSD-6xHis plasmids, were analyzed by immunofluorescence microscopy to visualize the localization of the fusion proteins. For each strain, micrographs are reported of the differential interference contrast (DIC), the immunofluorescence signal (AF555), the nuclear DNA staining (DAPI), and the merged image. Images are representative of three biological replicates for each yeast strain. Scale bar: 5 μm.

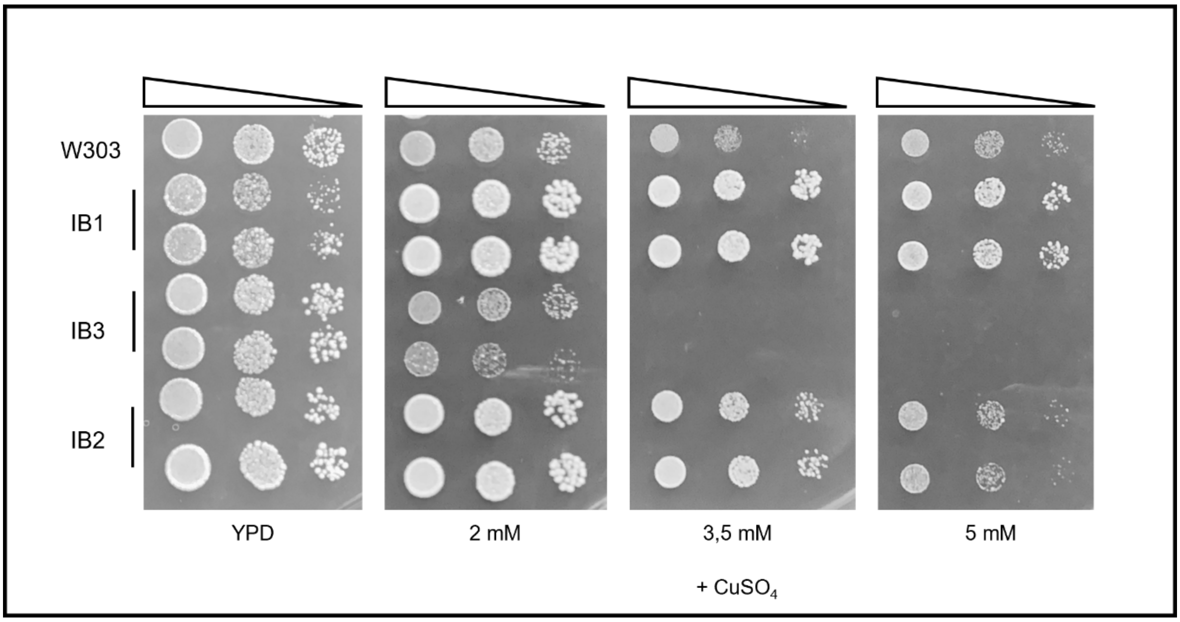

Figure S3. Copper sensitivity of yeast strains by viability assay. Growth and viability of the indicated strains (W303, IB1, IB2, IB3) were assayed in solid YPD medium containing increasing levels of $CuSO_4$ (2, 3.5, and 5 mM), as well in standard YPD medium as control. Images were captured after 3 days of incubation of the plates at 28°C. Three independent experiments were performed showing similar results.

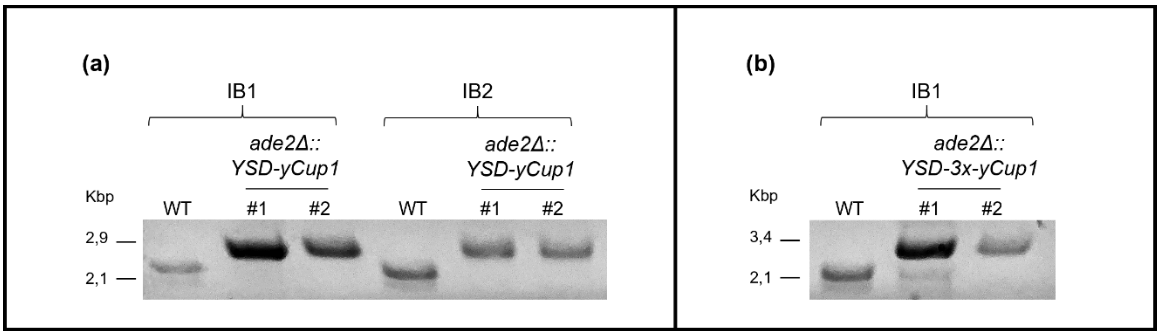

Figure S4. PCR analysis of *ADE2 locus*. Agarose gel electrophoresis of *ADE2 locus* PCR amplification using as template the genomic DNA extracted from two independent clones of the indicated strains, upon CRISPR/Cas9-assisted genome modification. Diagnostic primers (dgADE2, see Table ST3) were used, mapping outside the *ADE2* gene coding sequence. PCR size fragments are expected to be: ~2100 bp for parental (WT) allele; ~2900 bp for *ade2Δ::YSD-yCup1* allele (**panel a**); ~3350 bp for *ade2Δ::YSD-3x-yCup1* allele (**panel b**). Unmodified (WT) strains were used as controls.

Additional information on the *Saccharomyces cerevisiae* yeast strains.

Yeast (and bacterial) strains used in this work are listed in the **Table ST1**.

The yeast strains were considered as representative of either laboratory or natural (oenological) *Saccharomyces cerevisiae* strains. In particular, CENPK and W303 are yeast laboratory strains widely diffused in biomedical and biotechnological research centers. Additional information can be found in the Saccharomyces Genome Database (SGD, https://www.yeastgenome.org/). Moreover, eight natural yeast strains were used (namely, IB1 – IB8), belonging to the proprietary Collection of the Company "Italiana Biotecnologie Srl" (Italy), which have been isolated from grapes of different variety and geographic areas. Further characterization revealed strain-specific relevant features, as fermentative and/or aromatic properties, progressively supporting their use for the production at industrial level of either red or white wines. Notably, the genome of most Collection's strains had been fully sequenced, and several were deposited in DBVPG Collection (University of Perugia, Perugia, Italy), including IB1 – IB8. Additional information about the oenological strains used in this work is described in the following diagram.

Strain	***DBVPG ID***	***Origin (grape variety)***	***Geographic area***	***Country***	***Wine type***
IB1	#52 SF	*Ortrugo*	*Colli piacentini (PR)*	Italy	White
IB2	#62 SF	*Syrah*	*Rhone septentrional*	France	Red
IB3	#76 SF	*Cesanese*	*Piglio (FR)*	Italy	White
IB4	#73 SF	*Sangiovese*	*Montepulciano (SI))*	Italy	Red
IB5	#72 SF	*Cabernet sauvignon*	*Napa Valley (CA)*	USA	Red
IB6	#64 SF	*Barbera*	*Langhe piemontesi (CN)*	Italy	Red
IB7	#77 SF	*Sauvignon blanc*	*Collio friulano (UD)*	Italy	White
IB8	#78 SF	*Greco bianco*	*Avellino (AV)*	Italy	White

Additional Methods.

Yeast genomic DNA extraction and PCR amplification of *ADE2* locus.

Genomic DNA extraction from yeast cells was performed by the use of ZYMO RESEARCH (Quick-DNA Fungal/Bacterial Kits, D6005) columns; 1 μL of purified DNA was used as template for diagnostic PCR to amplify the *ADE2* locus with the specific dgADE2 primers (Table ST3).

Plasmidic DNA purification from bacteria.

Plasmidic DNA was purified from E.coli cells using the GenElute Plasmid Miniprep Kit (Sigma-Aldrich, cat. PLN350), following the manufacturer's instruction.

Yeast protein extraction.

Total proteins were extracted from the yeast cells using the trichloroacetic acid (TCA) method (1), with the following variations. 5 mL of yeast coltures (OD_{600}=1) were harvested and resuspended in 100 μL of 20% TCA. Cells were lysed using acid-washed glass beads (Sigma-Aldrich, cat. G8772) by alternating 30-second cycles (6000 rpm) in bead-beater (MagNA Lyzer, Roche) and 30-second cycles on ice. 500 μL of 10% TCA was then added to the samples and centrifuged for 10 min 14000 rpm. Pellet was then resuspended in 100 μL of 1X Laemmli Sample Buffer (2% sodium dodecyl sulfate, 10% Glycerol, Tris-HCl 62,5 mM pH 6.8, dithiothreitol 50 mM, bromophenol blue), incubated 5 min at 95°C, and after centrifugation the protein extracts in the supernatant were collected and used for Western blot assays.

Yeast Fluorescence microscopy.

Green fluorescence signals emitted by GFP-expressing yeast cells, were directly observed in living cells by confocal microscopy. Cells were collected after overnight growth in selective medium and resuspended in PBS solution. Cell suspension was dropped onto a slide (Superfrost, BDH) and covered with a coverslip. Yeast

cells were observed with a Leica SP5 confocal microscope using 63X HCX PL APO (NA 1.4) oil-immersion objectives. Imaging was performed at 1024 × 1024 pixels, with a 200 Hz acquisition rate. Images were processed using Fiji/ImageJ software.

Yeast copper sensitivity assay.

Yeast copper sensitivity was tested by viability assay as described (2), using solid YPD medium added with different concentrations of $CuSO_4$ (i.e., 2, 3.5, and 5 mM). Briefly, yeast cells were grown overnight at 28 °C in liquid medium, then the cultures were normalized to OD_{600}= 1, serially diluted (10-fold), and 5 µL drops were spotted on solid YPD medium containing copper, or without the metal as control. Plates were then incubated the at 28 °C for 3 days. Three independent experiments were performed.

Yeast genome editing by CRISPR/Cas9.

The CRISPR/Cas9 genetic manipulation of yeast cells was performed by the plasmid co-expressing the SpCas9 nuclease and the guide RNA molecule targeting the *ADE2* locus (gADE2-pMEL13-Cas9), generated as previously reported (2). The *ADE2* guide sequence (AATGCAATCAAATCTTTTCC) was designed accordingly to CRISpy-pop tool (available online, https://crispy-pop.glbrc.org/), and cloned into pMEL13-Cas9 by the In-Fusion Kit using the DNA fragment obtained by PCR amplification of the pMEL13-Cas9 backbone with the specific primers (6006-For; gADE target-Rev, Table ST3). The *ADE2* replacement in the yeast genome was achieved through standard co-transformation of the yeast cells with the gADE2-pMEL13-Cas9 plasmid (1 μg) and donor dsDNA molecules (3 μg), which were produced by PCR amplification of either pYSD-6xHis, pYSD-yCup1, or pYSD-3x-yCup1 plasmids, using the specific primers (respectively, ADE2-dsDNA IR For and Rev, Table ST3). Resulting dsDNA fragments consisted of 2718 bp (dsYSD-yCup1), or 3042 bp (dsYSD-3x-Cup1).

Plasmids Construction procedures.

The yeast plasmids listed in **Table ST2** (maps are in **Figure S1**) were obtained by sequential steps of molecular assembly performed using the In-Fusion HD Cloning Kit, following the manufacturer's instructions (Takara-bio, USA, Inc. (San Jose, CA, USA), Cat. 102518). The DNA fragments to be assembled were generated by PCR with the specific primers, whose sequence is reported in **Table ST3**.

1) The secretion signal MFα1 pre-pro-sequence (aa 1-85) was amplified from yeast (CENPK) genomic DNA with the YSD-alfa-FLAG-For and -Rev primers, and the PCR product was cloned in the multicopy pYES2 plasmid (Clontech), digested with HindIII-KpnI restriction enzymes and amplified with YSD-pYES-For and -Rev primers, generating the α1-FLAG-pYES plasmid.

2) The C-terminal region (aa 330-650) of the SAG1 α-agglutinin (containing the cell wall anchor domains) was amplified from yeast (CENPK) genomic DNA with the YSD-C-SAG-For and -Rev primers, and inserted at the 3'-end of MFα1-FLAG sequence by InFusion kit into the α1-FLAG-pYES plasmid, digested with XhoI-XbaI restriction enzymes and amplified with YSD-AlfaSAG-pYES-For and -Rev oligonucleotides, generating the α1-FLAG-SAG-pYES.

3) Between α1-FLAG and SAG, the coding sequence of three different *protein-of interest* has been inserted, maintaining the correct frame-shift: the yeast metallothionein (yCup1p), the mNeonGreen (GFP) fluorescent protein (3), and the exa-Histidine (6xHis) oligopeptide. The α1-FLAG-SAG-pYES plasmid was amplified by PCR with the YSD-neonGFP-pYES-For and -Rev primers, and resulting DNA fragment was joined to the coding sequence of either yCup1 (amplified from yeast CENPK genomic DNA with YSD-yCUP1-For and -Rev primers), the GFP (amplified from the pDRF1-GW-ymNeongreen-T2A-mTurquoise2 plasmid (Addgene # 125701) with the YSD-neonGFP-For and -Rev primers), or the exa-Histidine sequence (consisting of the annealing of the YSD-6His-For and -Rev oligonucleotides), respectively generating the pGAL-YSD-yCup1, pGAL-YSD-GFP, or the pGAL-YSD-6xHis.

4) The galactose-inducible *GAL1* promoter of pYES-derived plasmids was replaced by the GAP constitutive promoter from the *S.cerevisiae TDH3* gene, encoding the Glyceraldehyde-3-Phosphate Dehydrogenase isoform 3. The *TDH3* promoter sequence was amplified from yeast CENPK genomic DNA with the pYES-promGAP-For and -Rev primers, and the PCR product was joined with the DNA fragment amplified with the pYES-noprom-For and -Rev primers using either pGAL-YSD-yCup1, pGAL-YSD-GFP, or the pGAL-YSD-6xHis plasmids as template, finally generating the pGAP-YSD-yCup1, pGAP-YSD-GFP, or pGAP-YSD-6xHis plasmids.

5) The pYES2 nutritional marker (*URA3*) for the yeast selection was replaced by the NatR marker, conferring the resistence to the Nourseothricin antibiotic. The pGAP-YSD plasmids were linearized by NdeI digestion and amplified with pYES-deltaURA3-For and -Rev oligonucleotides, while the *NatR* gene was amplified from the pMEL15 plasmid (Addgene #107921) with the NrsR-For and -Rev primers. The PCR fragments were fused together by InFusion cloning kit, obtaining the pYSD-yCup1 and pYSD-6xHis plasmids.

6) The pYSD-YSD-3x-yCup1 plasmid, carrying three metallothionein proteins in tandem, was generated from the pYSD-yCup1 plasmid inserting two additional copies of the yCup1 coding sequence. The first copy was PCR-amplified from CENPK genomic DNA with the CUP1-sx-FOR and Rec-REV primers, and the second copy using the Rec-FOR and CUP1-dx-REV primers for the PCR amplification. Then, 1 μL of each PCR product was mixed and used as template in the PCR with the CUP1-sx-FOR and CUP1-dx REV primers. The resulting DNA fragment was cloned by the InFusion kit into the pYSD-yCup1 plasmid, digested with EcoRI-NotI restriction enzymes and amplified with pYES-3x-Cup-For and -Rev oligonucleotides, generating the final pYSD-YSD-3x-yCup1 plasmid.

Molecular assembly of the plasmids by InFusion HD reactions is schematically reported in the diagram below, where primers are indicated by numbers accordingly to **Table ST3**:

	**DNA fragment 1** *(PCR insert)*	*template*	*primers*	+	**DNA fragment 2** *(PCR linearized vector)*	*primers*	**InFusion HD reaction product** (*plasmid name*)
1	MFα1-FLAG	gDNA(#)	1, 2	+	pYES2	3, 4	α1-FLAG-pYES
2	SAG1 Cter	gDNA(#)	5, 6	+	α1-FLAG-pYES	7, 8	α1-FLAG-SAG-pYES
3	yCup1	gDNA(#)	13, 14	+	α1-FLAG-SAG-pYES	11, 12	pGAL-YSD-yCup1
	GFP	*pDRF1-…*	9, 10	+	α1-FLAG-SAG-pYES	11, 12	pGAL-YSD-GFP
	exa-Histidine		31, 32	+	α1-FLAG-SAG-pYES	11, 12	pGAL-YSD-6xHis
4	TDH3 (pGAP)	gDNA(#)	17, 18	+	pGAL-YSD-yCup1	15, 16	pGAP-YSD-yCup1
	TDH3 (pGAP)	gDNA(#)	17, 18	+	pGAL-YSD-GFP	15, 16	pGAP-YSD-GFP
	TDH3 (pGAP)	gDNA(#)	17, 18	+	pGAL-YSD-6xHis	15, 16	pGAP-YSD-6xHis
5	*NatR* marker	*pMEL15*	19, 20	+	pGAP-YSD-yCup1	35, 36	pYSD-yCup1
	NatR marker	*pMEL15*	19, 20	+	pGAP-YSD-6xHis	35, 36	pYSD-6xHis
6	yCup1 (2nd copy) a	gDNA(#)	27, 28				
	yCup1 (3rd copy) b	gDNA(#)	29, 30				
	yCup1 (2-3)	1 μL of each PCR(a,b) product	27, 30	+	pYSD-yCup1	33, 34	pYSD-3x-yCup1
	(#) CENPK genomic DNA						

All recombinant plasmids were extensively controlled by both DNA restriction analysis and Sanger sequencing.

Tables.

Table ST1. Yeast and bacterial strains.

	***S.cerevisiae* strain**	**Type**	**Source**	**Genotype**
1	CENPK (CEN.PK2-1C)	*Laboratory*	Euroscarf (#30000A)	*MAT*a, *ura3-52, trp1-289, leu2-3,112, his3Δ1, MAL2-8C, SUC2*
2	W303 (BMA64)	*Laboratory*	Euroscarf (#20000D)	*MATa/α, his3-11,15/his3-11,15; leu2-3,112/leu2-3,112; ura3-1/ura3-1; trp1Δ2/trp1Δ2; ade2-1/ade2-1; can1-100/can1-100*
3	IB1	*Natural*	Italiana Biotecnologie	*MATa/α*, Wild-type
4	IB1-YSD-yCup1	*Natural*	This study	*MATa/α, ade2Δ::YSD-yCup1*
5	IB1-YSD-3x-yCup1	*Natural*	This study	*MATa/α, ade2Δ::YSD-3x-yCup1*
6	IB2	*Natural*	Italiana Biotecnologie	*MATa/α*, Wild-type
7	IB2-YSD-yCup1	*Natural*	This study	*MATa/α, ade2Δ::YSD-yCup1*
8	IB3	*Natural*	Italiana Biotecnologie	*MATa/α*, Wild-type
9	IB4	*Natural*	Italiana Biotecnologie	*MATa/α*, Wild-type
10	IB5	*Natural*	Italiana Biotecnologie	*MATa/α*, Wild-type
11	IB6	*Natural*	Italiana Biotecnologie	*MATa/α*, Wild-type
12	IB7	*Natural*	Italiana Biotecnologie	*MATa/α*, Wild-type
13	IB8	*Natural*	Italiana Biotecnologie	*MATa/α*, Wild-type
	***E.coli* strain**			
14	StellaR		Clontech	F-, endA1, supE44, thi-1, recA1, relA1, gyrA96, phoA, Φ80d lacZΔ M15, Δ(lacZYA-argF) U169, Δ(mrr-hsdRMS-mcrBC), and ΔmcrA, λ-

Table ST2. List of the Plasmids generated in this study.

	Plasmid name	Length (bp)	Yeast marker	Promo ter	Transgene (N_{ter} – C_{ter})	size (aa)	MW (kDa)
1	MFα1-FLAG-pYES	6132	*URA3*	*GAL1*	α1–FLAG	85	12.7
2	MFα1-FLAG-SAG-pYES	7091	*URA3*	*GAL1*	α1–FLAG–SAG1-C_{ter}	440	46
3	pGAL-YSD-yCup1	7274	*URA3*	*GAL1*	α1–FLAG–yCup1–SAG1-C_{ter}	501	52.5
4	pGAL-YSD-GFP	7805	*URA3*	*GAL1*	α1–FLAG–GFP–SAG1-C_{ter}	678	72.8
5	pGAL-YSD-6xHis	7115	*URA3*	*GAL1*	α1–FLAG–6xHis–SAG1-C_{ter}	448	46.9
6	pGAP-YSD-yCup1	7566	*URA3*	*TDH3*	α1–FLAG–yCup1–SAG1-C_{ter}	501	52.5
7	pGAP-YSD-GFP	8094	*URA3*	*TDH3*	α1–FLAG–GFP–SAG1-C_{ter}	678	72.8
8	pGAP-YSD-6xHis	7341	*URA3*	*TDH3*	α1–FLAG–6xHis–SAG1-C_{ter}	448	46.9
9	pYSD-yCup1	7688	*NrsR*	*TDH3*	α1–FLAG–yCup1–SAG1-C_{ter}	501	52.5
10	pYSD-6xHis	7529	*NrsR*	*TDH3*	α1–FLAG–6xHis–SAG1-C_{ter}	448	46.9
11	pYSD-3x-yCup1	8141	*NrsR*	*TDH3*	α1–FLAG–3x-yCup1–SAG1-C_{ter}	652	68.8
12	gADE2-pMEL13-Cas9	10969	*KanR*	*SNR52*<br>*TEF1*	Guide RNA (*ADE2*)<br>SpCas9 endonuclease		

	**Additional plasmids**			**Origin**
13	pMEL13-Cas9	10969	*KanR*	*Ref* (2)
14	pMEL15	5966	*NrsR*	Addgene #107921
15	pDRF1-GW ymNeongreen-T2A-mTurquoise2	8479	*URA3*	Addgene #125701
16	pYES2	5856	*URA3*	Clontech

Table ST3. List of the Primers used in this study.

	Primer name	Sequence (5' – 3')
1	YSD-alfa-FLAG For	ACTATAGGGAATATTAAGCTTATGAGATTTCCTTCAATTTTTACTGCAGTTTTATTCGCAGCATCCTCCGC
2	YSD-alfa-FLAG Rev	TACTAGTGGATCCGAGCTCGGTACCCTTATCGTCGTCATCCTTGTAATCTTTATCCAAAGATACCCCTTCTTC
3	YSD-pYES For	CGAGCTCGGATCCACTAGTAACGGCCGC
4	YSD-pYES Rev	AGCTTAATATTCCCTATAGTGAGTCGTATTACAGC
5	YSD-C-SAG For	CTGGCGGCCGCTCGAGCTAGCGCCAAAAGCTCTTTTATC
6	YSD-C-SAG Rev	GATGCGGCCCTCTAGATTAGAATAGCAGGTACGACAAAAGC
7	YSD-AlfaSAG-pYES For	CTAGAGGGCCGCATCATGTAATTAGTTATGTCACGC
8	YSD-AlfaSAG-pYES Rev	TCGAGCGGCCGCCAGTGTGATGGATATCTGC
9	YSD-neonGFP For	CCGCCAGTGTGCTGGAATTATGGTCTCTAAGGGTGAAGAAG
10	YSD-neonGFP Rev	GATATCTGCAGAATTCCCTTGTACAATTCGTCCATACC
11	YSD-neonGFP-pYES For	AATTCTGCAGATATCCATCACACTGGCGGC
12	YSD-neonGFP-pYES Rev	CCAGCACACTGGCGGCCGTTACTAGTGG
13	YSD-yCUP1 For	CCGCCAGTGTGCTGGATTCAGCGAATTAATTAACTTCCAA
14	YSD-yCUP1 Rev	GATATCTGCAGAATTCCTTTCCCAGAGCAGCATGACT
15	pYES-noprom For	AAAAAACCCCGGATCGGACTAC
16	pYES-noprom Rev	TACTAGTGGATCATCCCCACGC
17	pYES-promGAP For	GATGATCCACTAGTATCCTTGATTACGTAAGGGAGTT
18	pYES-promGAP Rev	GATCCGGGGTTTTTTTTTTGTTTGTTTATGTGTGTTTATT
19	NrsR For	ATTTACTTATAATACCCCCGCCGGGTCACCCGGCCAGCGA
20	NrsR Rev	CATCGATAAGCTAGCGCAGGCATTTGCTCGGCATGCCGG
21	gADE target-Rev	TTCTAGCTCTAAAACGGAAAAGATTTGATTGCATTGATCATTTATCTTTCACTGCGGAGA
22	6006 For	GTTTTAGAGCTAGAAATAGCAAGTTAAAATAAGGCTAGTC
23	dgADE2 For	ACTCTTGTTGCAGGGCTACG
24	dgADE2 Rev	CTGACGTAGCGCTATCCTCG
25	ADE2-dsDNA IR For	TATAACAATCAAGAAAAACAAGAAAACCGGACAAAACAATCAAGTgctacagggcgcgtggggatgatccactag
26	ADE2-dsDNA IR Rev	CATTTTATATTATTTGCTGTGCAAGTATATCAATAAACTTATATAccgcgcgttggccgattcattaatgcaggg
27	CUP1-sx FOR	TCTGGGAAAGGAATTCTGACCGAGCTCGGATCCACTAGTAACGG
28	Rec REV	TTCATTCAGCTCCGGTTCCCAACGATCCTGCATAATTCCTTTCCCAGAGCA
29	Rec FOR	GATCGTTGGGAACCGGAGCTGAATGAATGTGCTGGATTCAGCGAATTAATT
30	CUP1-dx REV	CTAGCTCGAGCGGCCGCCAGTGTGATGGATATCTGCATAATTCCTT
31	YSD-6His For	CCGCCAGTGTGCTGGATCCCATCACCATCACCATCACGGAATTCTGCAGATATC
32	YSD-6His Rev	GATATCTGCAGAATTCCGTGATGGTGATGGTGATGGGATCCAGCACACTGGCGG
33	pYES-3x-Cup For	TCTGGGAAAGGAATTCTGACCGAGCTCGGATCCACTAGTAACGG
34	pYES-3x-Cup Rev	CTAGCTCGAGCGGCCGCCAGTGTGATGGATATCTGCATAATTCCTT
35	pYES-deltaURA3 For	GCTAGCTTATCGATGATAAG
36	pYES-deltaURA3 Rev	GTATTATAAGTAAATGCATGTA

Supplementary Material References

1) Wright, A. P. H., Bruns, M., & Hartley, B. S. (1989). Extraction and rapid inactivation of proteins from Saccharomyces cerevisiae by trichloroacetic acid precipitation. Yeast, 5(1), 51–53. https://doi.org/10.1002/yea.320050107

2) Granuzzo, S., Righetto, F., Peggion, C., Bosaro, M., Frizzarin, M., Antoniali, P., Sartori, G., & Lopreiato, R. (2023). Sulphate Uptake Plays a Major Role in the Production of Sulphur Dioxide by Yeast Cells during Oenological Fermentations. Fermentation, 9(3). https://doi.org/10.3390/fermentation9030280

3) Shaner, N. C., Lambert, G. G., Chammas, A., Ni, Y., Cranfill, P. J., Baird, M. A., Sell, B. R., Allen, J. R., Day, R. N., Israelsson, M., Davidson, M. W., & Wang, J. (2013). A bright monomeric green fluorescent protein derived from Branchiostoma lanceolatum. Nature Methods, 10(5), 407–409. https://doi.org/10.1038/nmeth.2413
